# Different rates of pollen and seed gene flow cause branch‐length and geographic cytonuclear discordance within Asian butternuts

**DOI:** 10.1111/nph.17564

**Published:** 2021-07-13

**Authors:** Lin‐Lin Xu, Rui‐Min Yu, Xin‐Rui Lin, Bo‐Wen Zhang, Nan Li, Kui Lin, Da‐Yong Zhang, Wei‐Ning Bai

**Affiliations:** ^1^ State Key Laboratory of Earth Surface Processes and Resource Ecology and Ministry of Education Key Laboratory for Biodiversity Science and Ecological Engineering College of Life Sciences Beijing Normal University Beijing 100875 China; ^2^ Centre for Individualised Infection Medicine (CiiM) & TWINCORE Joint ventures between the Helmholtz‐Centre for Infection Research (HZI) and the Hannover Medical School (MHH) Hannover 30625 Germany

**Keywords:** branch‐length discordance, gene flow, geographic discordance, introgression, whole‐genome resequencing

## Abstract

- Topological cytonuclear discordance is commonly observed in plant phylogenetic and phylogeographic studies, yet few studies have attempted to detect two other forms of cytonuclear discordance (branch length and geographical) and to uncover the causes of the discordance.
- We used the whole nuclear and chloroplast genome data from 80 individual Asian butternuts to reveal the pattern and processes of cytonuclear discordance.
- Our findings indicate that the chloroplast genome had substantially deeper divergence (branch‐length discordance) and a steeper cline in the contact zone (geographic discordance) compared with the nuclear genome. After various hypothesis have been tested, the results suggest that incomplete lineage sorting, positive selection and cytonuclear incompatibility are probably insufficient to explain this pattern. However, isolation‐by‐distance analysis and gene flow estimation point to a much higher level of gene flow by pollen compared with by seeds, which may have slowed down lineage divergence and mediated wider contact for nuclear genome compared with the chloroplast genome.
- Altogether, this study highlights a critical role of sex‐biased dispersal in causing discordance between the nuclear and plastid genome of Asian butternuts. Given its ubiquity among plants, asymmetric gene flow should be given a high priority in future studies of cytonuclear discordance.

## Introduction

Cytonuclear discordance refers to markedly different phylogenetic patterns between nuclear and organelle (chloroplast or mitochondria) markers, and is a common phenomenon in genealogical discordance (Rieseberg & Soltis, 1991; Toews & Brelsford, 2012). Generally, there are three forms of cytonuclear discordance, namely topological, branch length and geographic. Topological discordance is the most common, referring to the discrepancies of the branching structure between the organelle and nuclear genome (Rieseberg & Soltis, 1991). Branch‐length discordance involves cases in which the organelle genome is inferred to have substantially deeper divergence, whereas the nuclear genome shows relatively little or no divergence. This discordance is often reported in animals, such as birds (Hogner *et al*., 2012; Pavlova *et al*., 2013), amphibia (Firneno *et al*., 2020) and reptiles (Singhal & Moritz, 2012), but rarely in plants (but see Huang *et al*., 2014). Geographic discordance describes clines at nuclear and organellar markers in a hybrid zone that do not yield similar width and shape (Rita Di Candia & Routman, 2007; Renoult *et al*., 2009; Kindler *et al*., 2012). It is worth noting that even in cases when topological discordance does not occur, branch‐length and geographic discordance can still be observed and have non‐negligible influence on evolutionary history analyses. They indicate historical and demographic complexities in phylogeographic analysis and can prevent establishing conclusive taxonomies (Bonnet *et al*., 2017) and also bias the estimation of species divergence time with chloroplast DNA (Huang *et al*., 2014).

Several processes can cause cytonuclear discordance among closely related taxa. First, large effective population size (ancestral polymorphism) may lead to incomplete lineage sorting, such that phylogenetic relationships among chloroplasts or some nuclear markers fail to capture the true population histories (Funk & Omland, 2003). Second, positive selection on chloroplast and nuclear genomes, which results in fixation of different genomic parts, can be a critical process underlying cytonuclear discordance (Barrett & Schluter, 2008; Pavlova *et al*., 2013). Third, cytonuclear incompatibility could limit introgression of the nuclear genes interacting with organellar genomes, or of genes having adapted to local environments, whereas the rest of the nuclear genome could introgress freely (Takahata & Slatkin, 1984; Burton *et al*., 2013). Fourth, cytonuclear discordance may simply reflect sex‐biased gene flow in animals (e.g. Dai *et al*., 2013; Phuong *et al*., 2017; Sloan *et al*., 2017) and plants (Petit & Excoffier, 2009). Gene flow during the speciation process slows down species divergence, and higher levels of nuclear gene flow will lead to shallower divergence time or branch length of nuclear genomes compared with chloroplast genomes. Sometimes, deeper mitochondrial divergence compared with nuclear loci could be explained by a faster rate of molecular evolution of mitochondrial DNA, but this is not the situation for the chloroplast DNA because the chloroplast genome generally evolves between two‐fold and six‐fold more slowly compared with the nuclear genome (Wolfe *et al*., 1987; Drouin *et al*., 2008; Smith & Keeling, 2015), although there are exceptions to this rule (Williams *et al*., 2019).

Several attempts have been made to conduct comprehensive analysis of different mechanisms underlying cytonuclear discordance in animals. For instance, through comparing different mechanisms, Firneno *et al*. (2020) found that incomplete lineage sorting is the primary cause for discordance in Mesoamerican toads, whereas Singhal & Moritz (2012) concluded that introgression acting in concert with selection or sex‐biased dispersal caused discordance in lizards. For plants, studies have focused on cytoplasmic introgression or positive selection on chloroplast genome as principal causes of discordance (Renoult *et al*., 2009; Huang *et al*., 2014; Lee‐Yaw *et al*., 2019; Forsythe *et al*., 2020; Rose *et al*., 2021), whereas other mechanisms such as sex‐biased dispersal or cytonuclear incompatibility have not received as much attention. As is well known, the dispersal distance of pollen is generally at least an order of magnitude greater compared with that of seeds, especially in wind‐pollinated temperate trees (Petit *et al*., 2005). Therefore, it is imperative to make a comprehensive analysis on all possible processes including sex‐biased dispersal for a better understanding of cytonuclear discordance in plants.

Here, we used the Asian butternut (*Juglans* section *Cardiocaryon*), including three closely related species, *J. mandshurica*, *J. ailantifolia* and *J. cathayensis*. They are wind‐pollinated trees and produce heavy fruits of walnuts that fall in the vicinity of maternal plants. Compared with pollen dispersal by wind that may span several kilometres, seed dispersal of these species is extremely limited. In addition, only less than 1% of the fruits dispersed by scatter‐hoarding rodents can germinate under natural conditions (Ma *et al*., 2001, 2005). Therefore, Asian butternuts provide a unique opportunity to disentangle different processes underlying cytonuclear discordance, in particular, to highlight the importance of sex‐biased dispersal. Our previous study using nuclear microsatellites and chloroplast fragments had revealed geographic discordance in the contact zone between *J. mandshurica* and *J. cathayensis*, where chloroplast DNA generated a substantially steeper cline of admixture compared with nuclear DNA (Bai *et al*., 2016). Chloroplast DNA also gave a deep divergence time between the northern and southern lineages, but branch‐length discordance remains elusive due to the inherent problems associated with nuclear microsatellite data in dating lineage divergence (Selkoe & Toonen, 2006). However, facile hybridisation and morphological similarity among species of Asian butternuts point to recent nuclear divergence of them, leading us to also expect branch‐length discordance. Therefore, we consider that Asian butternuts represent an excellent system to reveal the pattern and process of cytonuclear discordance in plants.

For our purpose, we resequenced the nuclear and chloroplast genomes of 80 individual butternut trees throughout their ranges of distribution. The results confirmed the existence of both branch‐length and geographic cytonuclear discordance. Then, we conducted a comprehensive test of four possible processes underlying discordance including lineage sorting, positive selection, cytonuclear incompatibility and sex‐biased dispersal. Our findings highlight the importance of different rates between pollen and seed gene flow in the formation of cytonuclear discordance within Asian butternuts.

## Materials and Methods

### Sampling and sequencing

We collected leaf samples from 80 adult individuals throughout the whole range of Asian butternuts in northern and southern China, the Korean Peninsula, Japan, and Taiwan Island (Supporting Information Table S1). The total genomic DNA was extracted from dried leaf tissue using a plant total genomic DNA kit (Tiangen, Beijing, China). Whole‐genome resequencing using paired‐end libraries with an insert size of 350 bp was performed on Illumina HiSeq X‐ten instruments by NovoGene (Beijing, China), with read lengths of 150 bp on each end. Samples were sequenced to an average depth of 30×.

### Phylogenetic relationships and divergence time for the chloroplast genome

Reads from each individual Asian butternut and 15 individuals from 10 outgroup species were aligned to the chloroplast genome of *J. regia* NC_028617.1 (https://www.ncbi.nlm.nih.gov/nuccore/NC_028617.1/) by using Bwa v.0.7.12 (Li & Durbin, 2009). We removed the duplicate reads generated by PCR and performed single sample variant calling with Samtools v.1.3 (Li, 2011). The two inverted repeats were both excluded. For each position in the reference chloroplast genome, bases were called if the coverage was > 500 reads and if > 80% of the reads agreed with either the reference or an alternate base.

Using the annotation of NC_028617.1 chloroplast genome, 79 protein‐coding genes were aligned with Mafft v.7.017 (Katoh & Standley, 2013) and then converted to the protein‐coding sequence (CDS) alignment with Pal2nal v.14 (Suyama *et al*., 2006). We treated the 1^st^, 2^nd^ and 3^rd^ codon positions from each gene as different subsets, creating in total 3 × 79 = 237 subsets. We used PartitionFinder v.2.1 (Lanfear *et al*., 2017) to partition the data into subsets evolving at a similar rate and under the same nucleotide substitution model. The best partitioning scheme comprised three subsets with lengths of 15 268–24 038 bp.

Phylogenetic trees were reconstructed under Bayesian method using Beast v.2.4.8 (Bouckaert *et al*., 2014) based on the sequence alignments described previously. Divergence time was estimated using Beast with an uncorrelated lognormal relaxed clock and GTR+γ substitution model. A Yule process was specified as the tree prior. The ages of three fossils were used as minimum‐age calibration points; 40 million years ago (Ma) was used as the stem age of the ancestor of butternuts and black walnuts (Manchester, 1989), 40.4–48.6 Ma as the age of the *Oreomunnea–Alfaroa–Engelhardia* clade (Dilcher *et al*., 1976), and 84 Ma as the maximum root age of the phylogeny (Sims *et al*., 1998).

### Phylogenetic relationships and divergence time for nuclear genomes

The reads from each individual were mapped to the *J. mandshurica* reference genome (JMA_v.3.3.fasta, http://cmb.bnu.edu.cn/juglans/). SNPs were called and joined to create a multisample SNP dataset using the Sentieon Dnaseq v.201711.05 (Weber *et al*., 2016). The filtering strategy is according with (B. W. Zhang *et al*., 2019). After filtering and correction, a total of 10 693 416 SNPs remained. Linkage disequilibrium (LD) for each species was calculated using PopLDdecay v.3.40 (C. Zhang *et al*., 2019). To obtain neutral and independent SNPs, those located in CDS and its 20‐kb extension region were discarded and further thinned using a distance filter of 20 kb based on LD results (Fig. S1). To reduce false‐positive effects caused by sequencing error, singletons were excluded and the final dataset contained 2904 SNPs.

With the dataset of 2904 SNPs, we conducted population structure analysis using Structure v.2.3.4 (Pritchard *et al*., 2000) with the admixture model and uncorrelated allele frequencies. Markov Chain Monte Carlo analyses were run for 500 000 iterations after a burn‐in period of 200 000 iterations, and the number of clusters (*K*) was set to 1–5. The optimal value of *K* was determined using both StructureHarvester v.0.6.94 (Earl & vonHoldt, 2012) according to the delta *K* method of Evanno *et al*. (2005) and KFinder according to the parsimony method of Wang (2019). We also performed principal component analysis (PCA) using the R package snprelate v.1.6.2 (Zheng *et al*., 2012) with default settings.

We inferred species networks that model incomplete lineage sorting and gene flow using a maximum pseudo‐likelihood approach (Yu & Nakhleh, 2015) with the dataset of single‐copy nuclear genes. Species network searches were performed using PhyloNet v.3.7 (Than *et al*., 2008; Wen *et al*., 2018) with the command ‘InferNetwork_MPL’. We extracted 1622 single‐copy genes from the consensus genome and built ML gene trees with *J. olanchana* as the outgroup using RAxML v.8.2.8 (Stamatakis, 2014) with the GTR+γ model (see Notes S1). We performed network searches using only nodes in the rooted ML gene trees that had a bootstrap support of at least 80%, allowing for 0–3 reticulations with 25 runs for each and optimising the branch lengths and inheritance probabilities of the returned species networks under pseudo‐likelihood.

The analyses described so far provided strong evidence for gene flow between species of Asian butternuts; therefore, evaluating divergence time using strictly bifurcating tree methods was improper because gene flow can result in underestimates of species divergence time (Leache *et al*., 2014). We chose two methods to calculate the divergence time of nuclear genome. First, we used *fastsimcoal2* (Excoffier *et al*., 2013) to simulate the model inferred by PhyloNet (Fig. S2). To increase the power of genomic data, we relaxed the assumption of neutrality and obtained a dataset containing 23 750 SNPs using only a distance filter of 20 kb. Three‐dimensional joint site frequency spectra (3D‐SFS) were constructed with easySFS (https://github.com/isaacovercast/easySFS). We performed 100 000 coalescent simulations and computed log‐likelihoods based on simulated and observed 3D‐SFS matrixes. Global ML estimates for this model were obtained from 60 independent runs, with 50 conditional maximisation algorithm cycles. The mutation rate was set to 2.06 × 10^−9^ substitutions per site per year, and a generation time of 30 yr was assumed (Bai *et al*., 2018; B. W. Zhang *et al*., 2019). A parametric bootstrapping approach was used to construct 95% confidence intervals (CIs) with 100 independent runs for each bootstrap.

Second, we estimated divergence time using StarBeast2 (Ogilvie *et al*., 2017), eliminating admixed individuals with the *Q* value between 0.90 and 0.10. Because StarBeast2 cannot handle large amounts of data, we used 100 single‐copy nuclear genes for five pure individuals from each species. We used unlinked substitution models and set all sites to an HKY model. We linked the clocks for each locus under a strict molecular clock with a prior of substitution rate at 2.06 × 10^−9^ per site per year (Bai *et al*., 2018; B. W. Zhang *et al*., 2019). We used strict instead of relaxed clocks because strict clocks have been found to be sufficient for closely related species (Ogilvie *et al*., 2017). We ran the MCMC chains for each analysis for 0.5 billion generations, sampling every 50 000, and checked that the chains had proper mixing and convergence with Tracer v.1.6.0 (Rambaut *et al*., 2014), confirming an ESS of over 200 for every parameter including tree topology.

### Population demographic analysis

Analysis of demographic history for all three species was conducted using the pairwise sequentially Markovian coalescent (PSMC) model, which estimates population size changes utilising information from the whole genome of a single diploid individual (Li & Durbin, 2011). The reads from each individual were mapped to the *J. mandshurica* reference genome. The analysis command included the options ‘‐N25’ for the number of cycles of the algorithm, ‘‐t15’ as the upper limit for the most recent common ancestor, ‘‐r5’ for the initial θ/ρ, and ‘‐p 4 + 25*2 + 4 + 6’ atomic intervals. The reconstructed population history was plotted using R script with the substitution rate ‘‐u 2.06e‐9’ and a generation time of 30 yr (Bai *et al*., 2018). To determine variance in the estimated effective population size, we performed 100 bootstraps for each species.

### Chloroplast selection analysis

Several tests were used to look for a molecular signature of selection on chloroplast genes. Considering that these tests generally require a modest to high amount of variation between taxa, we restricted tests of selection to 12 chloroplast genes that had at least two variable sites across full dataset.

First, we performed tests with branch models in codeml executed in Paml v.4.9d (Yang, 1997). We used three models in the branch test: (a) a 1‐ratio test, a single, global ω (*K*_a_ */K*_s_) for all branches; (b) a 2‐ratio test, ω = 1 for the branches of interest; and (c) a 2‐ratio test, different values of ω for the branches of interest. If model (c) performed better than models (a) and (b), the branches of interest would be under positive selection.

Second, we used Tajima's *D* (Tajima, 1989) and Fu and Li’s *F* and *D* (Fu & Li, 1993; Fu, 1997) to test the two main chloroplast clades for deviations from neutral evolution. Test statistics were calculated separately for each gene using Arlequin v.3.5.2.2 (Excoffier *et al*., 2005) and compared these with 5000 simulated samples to test for significance.

Third, we used the McDonald–Kreitman tests (MKTs) (McDonald & Kreitman, 1991), which involve counting the number of nonsynonymous to synonymous polymorphisms within a focal group and between this group and an outgroup to test positive selection. MKTs were run using python script (see Notes S2), which uses Fisher’s exact tests to assess statistical significance.

### Signatures of selection in nuclear genome

Because outlier loci identified by a single method may generate false positives (Lotterhos & Whitlock, 2015), we used three genome scan methods to detect signatures of selection: one linkage disequilibrium‐based (G12), one site frequency spectrum‐based (CLR) and one population differentiation‐based methods (*F*_ST_).

G12 examines a symmetric window of a fixed number of SNPs around each focal SNP using unphased multilocus genotypes data (Harris *et al*., 2018). We used a window size of 400 SNPs to calculate G12 values with python script (https://github.com/ngarud/SelectionHapStats) using the default parameters setting.

The test of composite likelihood ratio (CLR) quantified deviations of allele frequency spectrum relative to the global observed patterns (Nielsen *et al*., 2005). We performed the CLR test in 20‐kb stepping windows using SweepFinder2 (DeGiorgio *et al*., 2016) with the default parameters setting. We used Tajima's *D* (Tajima, 1989) to test the two main clades for changes from neutral evolution in 20‐kb stepping windows.

We computed estimates of *F*_ST_ in 20‐kb stepping windows using Vcftools v.0.1.13 (Danecek *et al*., 2011). Regions with *F*_ST_ falling in the top 1% were designated as outliers. Because genomic regions with reduced recombination rate are predicted to have increased genetic divergence between species and may harbour alleles under selection, we estimated a scaled population recombination rate, ρ = 4*N*_e_ *r*, using LDhat v.2.2 (McVean *et al*., 2004; Auton & McVean, 2007). The rjMCMC chain used a burn‐in of 100 000 iterations followed by 2 000 000 iterations, sampling every 5000^th^ iteration, and using a block penalty of 5.

### Cytonuclear incompatibility analysis

We hypothesised that SNPs from nuclear‐encoded cytonuclear interacting protein genes could have a similar population structure to the chloroplast compared with the nuclear genome. The list of nuclear‐encoded cytonuclear interacting protein genes in *Arabidopsis thaliana* was obtained from Cytonuclear Molecular Interactions Reference for Arabidopsis (CyMIRA) (Forsythe *et al*., 2019). Based on CyMIRA, we obtained a total of 535 mitochondrial, 293 chloroplast and 82 dual targeted genes, and then extracted the corresponding longest protein sequences from Araport11 (Cheng *et al*., 2017). All protein sequences of Asian butternuts were aligned to the nucleocytoplasmic interaction protein sequences of *A. thaliana* with a Blastp *e*‐value < 10^−5^ (Camacho *et al*., 2009). Subsequently, MCScan was executed to detect orthologous gene pairs (*C*‐score > 0.7) using the Blastp output and annotation file as its inputs. Finally, we obtained the SNPs of these cytonuclear interacting genes within Asian butternuts, conducted Structure analysis and constructed a ML tree using the methods mentioned above. Considering that only a few SNPs from several genes may be active rather than all the genes involved in the interaction, we calculated *F*_ST_ for each polymorphic site of each orthologous gene to see whether their *F*_ST_ values were nearly close to chloroplast *F*_ST_.

### Estimates of pollen and seed gene flow

To estimate historical gene flow between the two clades, we used the software Migrate v.4.4.3 (Beerli, 2006) to estimate the effective number of migrants (4*Nm*, where *N* is the effective population size and *m* is the migration rate per generation) with nuclear and chloroplast data, assuming a migration matrix model with symmetric migration rates and different population sizes. Priors for the mutation‐scaled migration rate *M* (= *m*/μ) and population size θ (= 4*N*μ for nuclear DNA and *N*μ for chloroplast DNA, where μ is the mutation rate per generation) were both uniform with a range 0–10 000 (mean 5000) and 0–0.01 (mean 0.005), respectively. We ran four concurrent chains, including one long chain (1 000 000 trees) with temperatures of 1, 1.5, 3 and 10^6^ and 10 000 trees discarded as initial ‘burn‐in’ and sampling increment as 100.

### Isolation by distance (IBD) and isolation by environment (IBE)

We did IBD analysis for both chloroplast and nuclear data for all the individuals and admixed individuals. We used a stratified Mantel test in which the permutation scheme is changed to permute the locations of individuals within each putative cluster: a Mantel test with 10 000 random permutations was performed between the matrix of pairwise *D_xy_* and that of the geographic distances, using the package vegan (Oksanen *et al*., 2018) in R v.4.0‐0.

To investigate the role of environmental factors in shaping the spatial genetic differentiation, we calculated IBE analysis. We performed Gradient forest (Ellis *et al*., 2012) analyses to identify the environmental variables (Table S2) that best explained the distribution of nuclear genetic diversity (see Notes S3) and then calculated pairwise Euclidean environmental differences between sites using these variables (Fig. S3). Second, we used partial Mantel test controlling for geographic distance to assess associations between pairwise *D_xy_* and environmental distance with significance determined using 10 000 permutations in the package vegan.

## Results

### Phylogenetic relationships and divergence time for the chloroplast genome

**Fig. 1:**
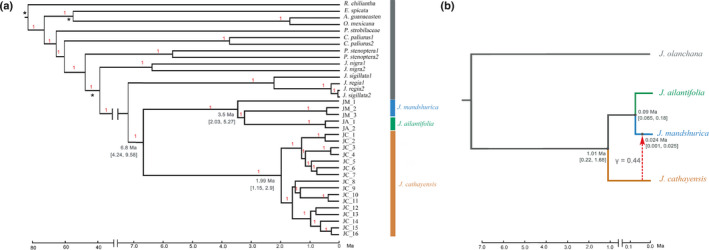
(a) Beast **‐**derived chronograms of 21 haplotypes of Asian butternuts (*Juglans mandshurica*, *J. ailantifolia*, and *J. cathayensis*) based on the chloroplast genome. Posterior probabilities (>0.95) are labelled on each node. The ages of three fossils were used as minimum‐age calibration points with an asterisk and blue bars, and the numbers below the bars indicate the 95% HPD of time estimates in Ma. (b) Most supported species network of the 1622 single‐copy orthologous nuclear gene dataset inferred with PhyloNet. The dotted line indicates an introgression and the number next to the red dotted line indicates inheritance probabilities. The numbers near the nodes indicate the 95% CI of divergence time estimates using *fastsimcoal2* simulation.

Our reference‐guided alignment approach recovered a minimum of 150 931 bp (coverage rate of 99%) of the chloroplast genome for each sample. Excluding indels, a total of 56 substitution polymorphisms identified a total of 21 haplotypes (Fig. 1a). Overall, two main clades were recovered, one associated with *J. mandshurica* and *J. ailantifolia* (northern clade, five haplotypes) and one associated with *J. cathayensis* (southern clade, 16 haplotypes). A point estimate of the divergence time between the two clades was dated to 6.80 Ma (95% highest posterior density (HPD): 4.24–9.58 Ma); the divergence time for all 16 haplotypes of *J. cathayensis* was 1.99 Ma (95% HPD: 1.15–2.9 Ma), and that for five haplotypes of *J. mandshurica* and *J*. *ailantifolia* was 3.50 Ma (95% HPD: 2.03–5.27 Ma, Fig. 1a). The mutation rates for the three subsets by the best partitioning scheme were 2.88 × 10^−11^, 2.10 × 10^−11^ and 1.04 × 10^−10^ per site per year, respectively.

### Phylogenetic relationships and divergence time for the nuclear genome

**Fig. 2:**
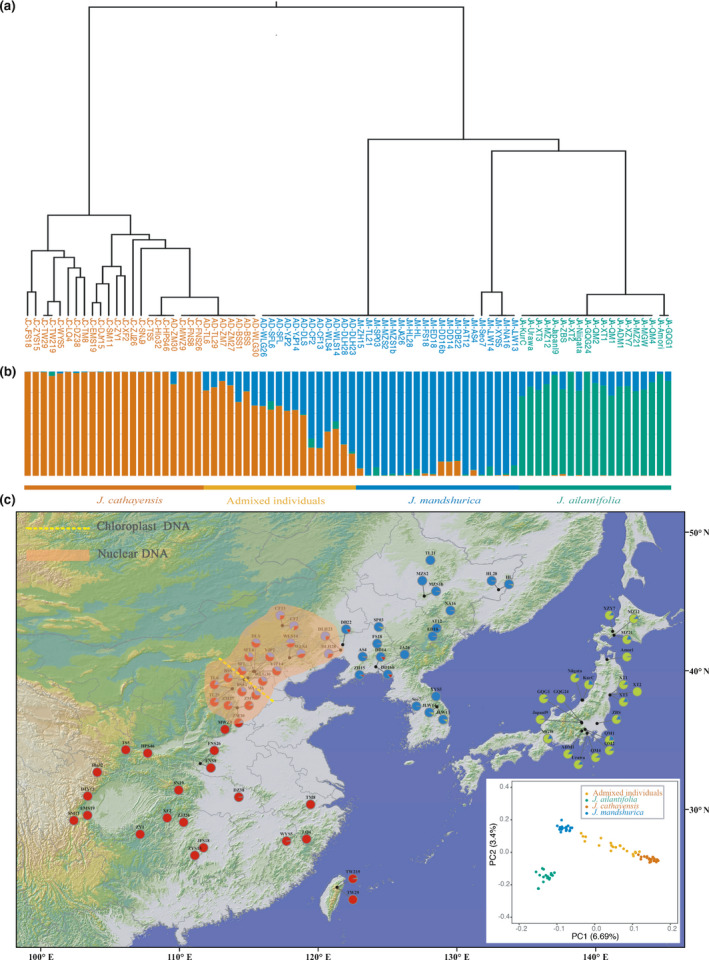
(a) Chloroplast phylogeny of the 80 individual Asian butternuts (*Juglans mandshurica*, *J. ailantifolia* and *J. cathayensis*). (b) Histograms of the Structure assignment test for 80 individuals of Asian butternuts based on the SNPs of the whole nuclear genome. (c) Geographic origin and PCA of the 80 individual Asian butternuts and their colour‐coded grouping at the most likely *K* = 3.

Aligning the cleaned reads to the *J. mandshurica* reference genome resulted in 33× vertical depth and 95% horizontal genome coverage (Table S1). After filtering and correction, 2904 SNPs remained and were used to conduct the analysis by Structure and PCA. Using the parsimony method of Wang (2019), *K* = 3 was found to be the optimal number, although the delta*K* method supported *K* = 2 as the most favourable. For *K* = 2, individuals of *J. mandshurica* and *J. ailantifolia* were clustered into one group and *J. cathayensis* into the other group; by contrast, for *K* = 3, individuals of each species were clustered into their respective groups. Whether *K* was two or three, 20 individuals from the contact zone between *J. mandshurica* and *J. cathayensis* exhibited a mixed ancestry, forming a continuous cline (Fig. 2b). However, these 20 individuals harboured only two chloroplast haplotypes (JM_1, JC_11), exhibiting a steep cline (Fig. 2a). Three species‐specific clusters were also identified by the PCA of nuclear SNPs (Fig. 2c), with the first two components explaining 6.69% and 3.40% of the total variance, respectively.

PhyloNet analysis was executed on 1622 independent single‐copy nuclear genes, and the results were used to sort the samples into three major clades, corresponding to *J. cathayensis*, *J*. *mandshurica*, and *J. ailantifolia*. Topology allowing one past hybridisation event is the best, in which *J. mandshurica* was introgressed by *J. cathayensis* (Fig. 1b). The inheritance probabilities showed that *J. mandshurica* had a genomic contribution of 44% from *J. cathayensis*.

When using *fastsimcoal2* to simulate the model inferred by PhyloNet (Fig. 1b), the divergence time between the northern clade and southern clade was only 1.01 Ma (95% CI: 0.22–1.68 Ma), and that between *J. mandshurica* and *J. ailantifolia* was 0.09 Ma (95% CI: 0.065–0.18 Ma) (Fig. S2). When using StarBeast2, the divergence time between the two clades was 0.38 Ma (95% HPD: 0.33–0.43 Ma), and that between *J. mandshurica* and *J. ailantifolia* was dated to 0.14 Ma (95% HPD: 0.09–0.19 Ma) (Fig. S4). A much smaller divergence time between the northern and southern clade in StarBeast2 than in *fastsimcoal2* accords well with the theoretical prediction of Leache *et al*. (2014) that gene flow between nonsister species results in underestimated speciation times for species involved in hybridisation. Regardless of whichever estimate being used, the divergence times for nuclear genome were substantially lower than that inferred from the chloroplast genome.

### Small effective population size since the early Pleistocene

**Fig. 3:**
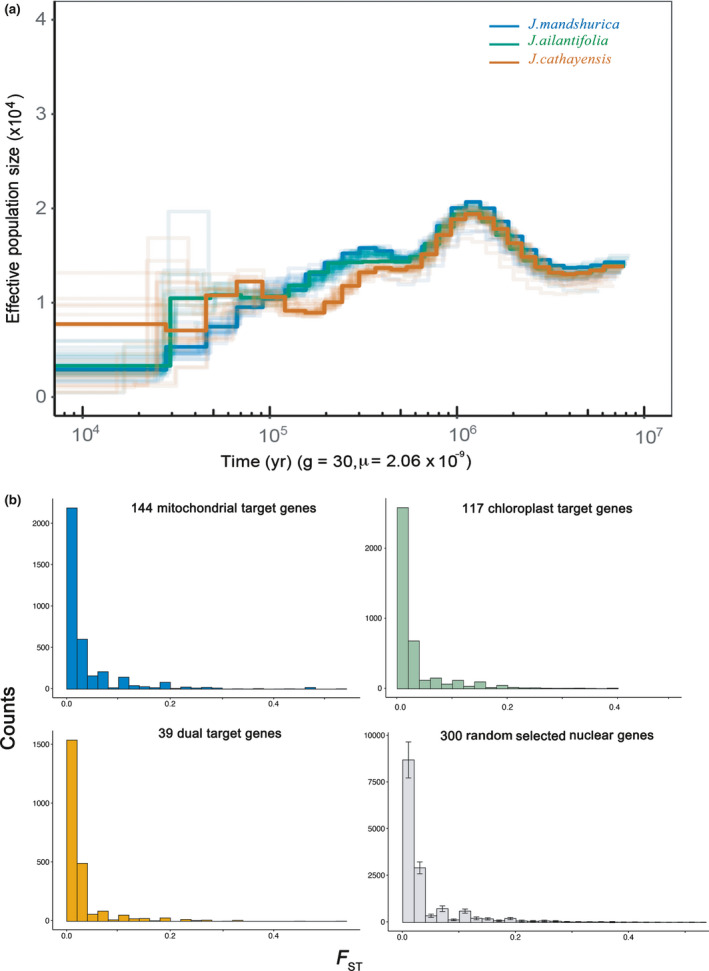
(a) Pairwise sequentially Markovian coalescent (PSMC) estimates of the changes in effective population size over time for Asian butternuts (*Juglans mandshurica*, *J. ailantifolia* and *J. cathayensis*). (b) *F*_ST_ for each polymorphic site of 144 mitochondrial, 117 chloroplast, 39 dual targeted nuclear‐encoded protein‐coding genes and 300 random selected nuclear genes without interaction with organelle. Error bar in panel (b) is mean ± SD.

During the period from 6.0 to 0.5 Ma (mid‐Pleistocene), the similarity between the *N*_e_ dynamics of the three species was pronounced (Fig. 3a). After 0.5 Ma, the *N*_e_ of *J*. *cathayensis* declined rapidly and its history of change deviated from those of the other two species. The *N*_e_ of *J*. *mandshurica* and *J*. *ailantifolia* remained relatively stable between 0.5 and 0.1 Ma, but *J*. *mandshurica* declined faster than *J*. *ailantifolia* during 0.1 and 0.03 Ma. Between 25 and 10 ka, *J*. *mandshurica* and *J*. *ailantifolia* decreased to their smallest population size (*N*_e_ ≈ 0.3 × 10^4^), lower than *J. cathayensis* (*N*_e_ ≈ 0.8 × 10^4^). The finding of consistently small effective population size coupled with long geographic isolation by the arid belt between the northern and southern clade (Bai *et al*., 2016) implied that topological cytonuclear discordance is unlikely to occur in Asian butternuts.

### No evidence for positive selection on the chloroplast genome

For the 12 genes with two more variations across the full dataset, models of separate ω values for the two main chloroplast clades (model c) did not perform better than the model of a single global ω (model a) or the model with ω = 1 for the branch of interest (model b). This indicated that all tested chloroplast genes had evolved in a neutral (ω = 1) or nearly neutral fashion (e.g. via purifying selection with ω < 1) (Table 1). Tajima's *D* were not significantly negative for all the 12 genes within each clade, but was significantly positive for *ccsA* within the southern clade. Fu & Li's *D* and *F* was significantly negative only for *ycf1* within northern clade (Table 1). MKT tests did not show any significance for all 12 genes for both clades. Taken together, these results suggest that nearly all chloroplast genes had neutrally evolved or were under purifying selection, and only *ycf1* may have been subject to positive selection, which codes for a protein of *c*. 1800 amino acids (Dong *et al*., 2015).

**Table 1:** Genetic variation in selected chloroplast genes of Asian butternuts and results from molecular tests of selection at the clade and gene level.

Gene	Diversity		Neutrality tests			MK‐tests	paml branch tests			
	No. of polymorphic sites across full dataset	Haplotype	Tajima's *D*	Fu & Li's *D* ^a^	Fu & Li's *F* ^a^	*P*‐value	Global ω	H0: likelihood (global ω)	H1: likelihood (ω = 1)	H2: likelihood (diff ω)
*ycf1* (5676 bp)										
South Clade	11	9	−1.69	**−2.53**	**−2.46**	0.47	0.41	−809.92	−809.92	−809.92
North Clade	5	4	1.46	1.10	1.34	0.085	0.41	−809.92	−809.92	−809.92
*ndhF* (2226 bp)										
South Clade	4	4	−1.58	−1.96	−1.99	1.00	0.21	−3253.23	−3252.52	−3252.72
North Clade	0	1	na	na	na	0.81	0.21	−3253.23	−3253.23	−3253.23
*matK* (1518 bp)										
South Clade	2	3	−1.01	−0.73	−0.86	0.74	0.36	−2021.9	−2021.01	−2022.58
North Clade	0	1	na	na	na	0.67	0.36	−2021.9	−2021.9	−2021.9
*ndhD* (1521 bp)										
South Clade	2	3	0.31	−0.73	−0.47	0.55	0.46	−2042.77	−2042.77	−2042.77
North Clade	0	1	na	na	na	1.00	0.46	−2042.77	−2042.77	−2042.77
*ccsA* (957 bp)										
South Clade	1	2	−1.15	−1.67	−1.63	1.00	1.39	−1275.52	−1275.52	−1275.52
North Clade	2	2	**2.28**	0.75	1.32	0.67	1.39	−1275.52	−1275.52	−1275.52
*ndhA* (1095 bp)										
South Clade	1	2	−1.15	−1.67	−1.63	0.76	0.40	−1535.59	−1535.59	−1535.59
North Clade	1	2	1.72	0.54	0.96	0.76	0.40	−1535.59	−1535.59	−1535.59
*ndhH* (1182 bp)										
South Clade	1	2	−1.15	−1.67	−1.63	0.50	0.09	−1675.34	−1673.86	−1674.11
North Clade	1	2	−0.25	0.54	0.34	1.00	0.09	−1675.34	−1675.34	−1675.34
*rpoB* (3213 bp)										
South Clade	1	2	−1.15	−1.67	−1.63	0.83	0.20	−4553.59	−4553.53	−4554.29
North Clade	0	1	na	na	na	na	0.20	−4553.59	−4553.59	−4553.59
*atpI* (744 bp)										
South Clade	0	1	na	na	na	na	0.49	−1021.61	−1021.09	−1021.35
North Clade	1	2	−1.10	−1.85	−1.79	na	0.49	−1021.61	−1021.61	−1021.61
*rpl14* (369 bp)										
South Clade	0	1	na	na	na	na	0.21	−502.94	−502.94	−502.94
North Clade	1	2	1.53	0.54	0.91	1.00	0.21	−502.94	−502.94	−502.94
*rpoC2* (4197 bp)										
South Clade	0	1	na	na	na	0.73	0.30	−5977.21	−5977.04	−5977.13
North Clade	0	1	na	na	na	0.67	0.30	−5977.21	−5976.5	−5977.98
*rpoA* (987 bp)										
South Clade	0	1	na	na	na	na	0.46	−1359.84	−1359.44	−1359.64
North Clade	0	1	na	na	na	na	0.46	−1359.84	−1359.02	−1359.41

MK‐tests, McDonald–Kreitman tests with *Juglans nigra* as the outgroup; na, not applicable.

^a^ Significant values are in bold and for paml tests are based on likelihood ratio tests as follows: H1 compared with H0, H2 compared with H0.

### Signatures of selection in nuclear genome

By applying the G12 approach, we identified a total of 1011 regions falling into the 1% top values for *J. mandshurica*, associated with 491 candidate genes, and for *J. cathayensis*, we also identified 1011 regions associated with 496 genes. The CLR test detected a total of 292 genomic regions falling into the 1% top values, harbouring 98 candidate genes for *J. mandshurica* and 292 genomic regions harbouring 105 genes for *J. cathayensis*. Tajima’s *D* was significantly negative for 479 and 1038 regions respectively for *J. mandshurica* and *J. cathayensis*. Mean *F*_ST_ across the genome and falling into the 1% top values were 0.098 ± 0.066 and 0.419 ± 0.085, respectively. There were 243 regions over the 1% top values in the comparison between *J. mandshurica* and *J. cathayensis*, associated with 255 candidate genes for both species.

**Fig. 4:**
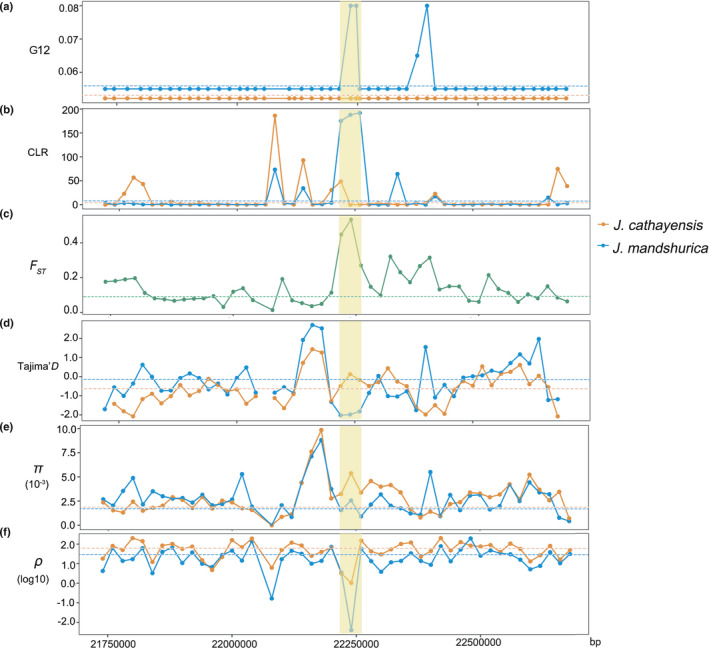
Scanning for signals of selection on nuclear genome of *Juglans mandshurica* and *J. cathayensis*. (a) G12 statistics (G12); (b) composite likelihood ratio (CLR); (c) *F*_ST_; (d) Tajima's *D*; (e) average nucleotide diversity (π); (f) recombination rate (ρ). The *x*‐axis shows the position on chromosome 7 and the *y*‐axis shows the values of each analyses. The light orange shadow indicates the two regions under selection on nuclear genome and the horizontal dotted lines indicate genome‐wide level of the summary statistics.

To reduce the rate of false positives, we combined multiple statistics of the aforementioned three methods and obtained two adjacent regions (Chr7 22220000, 22240000) associated with two genes (JMA026091, JMA025840) under selection (Fig. 4). Tajima's *D* values are significantly negative for the two genes in *J. mandshurica*, −2.018 and −1.980 (*P* < 0.05), but not in *J. cathayensis*, −0.490 and 0.126 (Fig. 4d) (*P* > 0.05). The population recombination rate (ρ = 4*N*_e_ *r*) and nuclear diversity of *J. mandshurica* in the two regions are much lower than the adjacent regions (Fig. 4e,f), but *J. cathayensis* did not show the same pattern (Fig. 4e,f). Taken together, the two genes are under positive selection are in *J. mandshurica*, but not in *J. cathayensis*. JMA026091 is a *TVP38/TMEM64* family membrane protein encoding genes linked with the shell thickness of walnut in *J. regia* (Bernard *et al*., 2021). The function of JMA025840 is unknown but, considering it is adjacent to JMA026091, we suspected that JMA025840 is actually under linked selection.

### No cytonuclear incompatibilities between chloroplast and nuclear genomes

According to CyMIRA, 535 mitochondrial, 293 chloroplast and 82 dual targeted genes were involved in direct cytonuclear interaction for *Arabidopsis thaliana*. In our dataset, we finally identified 144 mitochondrial, 117 chloroplast and 39 dual targeted genes (Table S3). Subsequently, 5367, 6023 and 3555 SNPs were obtained for mitochondrial, chloroplast and dual targeted genes of all the individuals, respectively. Structure analyses of these SNPs showed a similar population structure to the nuclear genome rather than to the chloroplast genome (Figs. S5, S6). Additionally, *F*_ST_ for each polymorphic site for each gene were different from the chloroplast *F*_ST_, but similar to the *F*_ST_ of randomly selected nuclear genes (Fig. 3b).

### Different rates of pollen and seed gene flow

For nuclear genome data, estimates of gene flow (4*Nm*) were moderate to high: from the northern group to the southern group; there were 3.716 migrants per generation (95% HPD: 3.437–3.892) and 37.91 (95% HPD: 37.22–38.14) for the opposite direction. For the northern group and the southern group, θ (4*N*μ) values were 0.0013 and 0.0019, respectively. For chloroplast genome data, estimates of gene flow (*Nm*) were low, from the northern group to the southern group being 0.416 migrants per generation (95% HPD: 0.235–0.650), and that of the opposite direction being 0.400 (95% HPD: 0.203–0.637). For the northern group and the southern group, chloroplast θ (*N*μ) values were 0.00018 and 0.00017, respectively.

### IBD and IBE

**Fig. 5:**
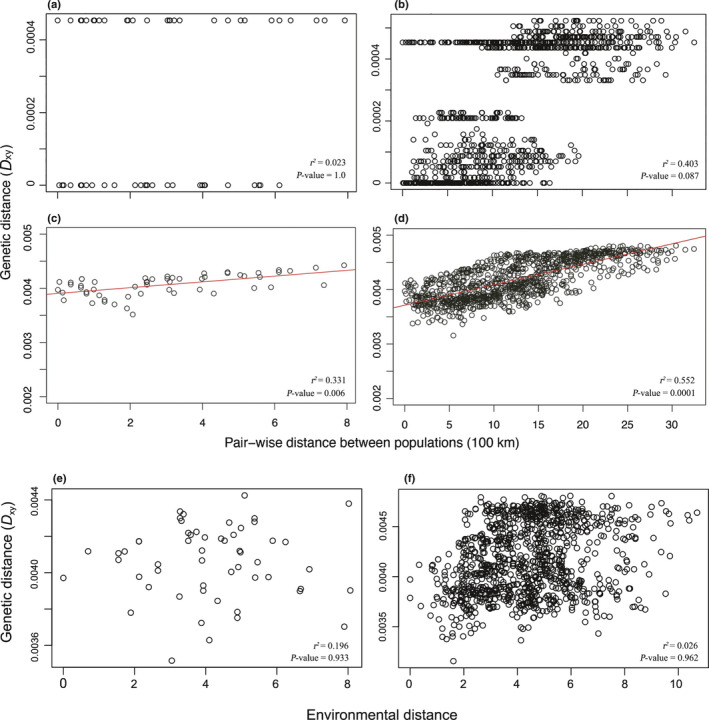
Pairwise genetic distance for chloroplast DNA of *Juglans mandshurica* and *J. cathayensis* are regressed onto geographic distance between admixed individuals (a) and all individuals (b) and for nuclear DNA between admixed individuals (c) and all individuals (d). Pairwise genetic distance for nuclear DNA are regressed onto environmental distance between admixed individuals (e) and all individuals (f). The red lines in panels (c) and (d) indicate linear regression.

A stratified Mantel test for chloroplast data indicated no significant correlation between genetic distance and geographic distance both for admixed individuals (*r* ^2^ = 0.023, *P* = 1.0; Fig. 5a) and all the individuals (*r* ^2^ = 0.403, *P* = 0.087; Fig. 5b), presumably due to an extremely low seed gene flow. By contrast, there was a significant correlation with nuclear data both for admixed individuals (*r* ^2^ = 0.331, *P* = 0.006; Fig. 5c) and for all the individuals (*r* ^2^ = 0.552, *P* = 0.0001; Fig. 5d).

After controlling for geographic distance, we found no significant pattern of IBE based on distance derived from environmental changes for both admixed individuals (*r* ^2^ = 0.196, *P* = 0.933; Fig. 5e) and all the individuals (*r* ^2^ = 0.026, *P* = 0.962; Fig. 5f).

## Discussion

Compared with the nuclear genome, the chloroplast genome is uniparentally inherited, generally without recombination, and has a slower evolutionary rate, except for a few genes in several lineages (Jansen *et al*., 2007; Williams *et al*., 2019). Despite mechanisms such as chloroplast–nuclear functional compensation that tie chloroplast and nuclear machinery to the same evolutionary path (Burton *et al*., 2013), more systems have been found to have inconsistent genetic patterns between chloroplast and nuclear DNA. In this study, we report an underrepresented example of cytonuclear discordance in plants, not only in terms of branch lengths but also geographical distributions. After examining various hypotheses, we provide strong supporting evidence for the importance of highly different pollen and seed flow in causing cytonuclear discordance.

### Branch‐length and geographic cytonuclear discordance among Asian butternuts

Our results are consistent with findings by Bai *et al*. (2016) in that two major chloroplast clades were found, one for *J. cathayensis* and the other for *J. mandshurica* and *J. ailantifolia* (Fig. 1a). The divergence time between these two clades was estimated as *c*. 6.38 Ma, much earlier than that revealed by *fastsimcoal2* analysis of nuclear genomes, that is 1.01 Ma (Fig. 1b). Furthermore, this pattern still holds when estimated with the coalescence approach (StarBeast2) for nuclear genomes, which yields an even shallower divergent time of 0.38 Ma (Fig. S4). It is well known that coalescent‐based methods assuming no postdivergence gene flow will usually underestimate speciation time (Leache *et al*., 2014). Therefore, a strong pattern of branch‐length discordance is revealed among Asian butternuts. Similar branch‐length discordance was reported for two closely related balsam poplars (Huang *et al*., 2014).

Both chloroplast and nuclear genomes clearly divided Asian butternuts into two clades, but the boundary location and width between regions were different between the two genomes (Fig. 2). Chloroplast genomes formed a dividing line for which individuals with different haplotypes of different clades were only found in one population, while the nuclear genome formed a much wider contact zone in which admixed individuals were found in a wider range of populations. Therefore, in addition to branch‐length discordance, there are also geographic cytonuclear discordance for Asian butternuts.

Similar phylogeographic breaks in northern China have been also demonstrated for several other species, including *Acer mono*, *Populus davidiana* and *Lindera obtusifolia* (Guo *et al*., 2014; Liu *et al*., 2014; Zong *et al*., 2015; Bai *et al*., 2016; Ye *et al*., 2017; Song *et al*., 2020). The breaks are generally attributed to the climatic barrier of the arid belt since the late Miocene (Milne & Abbott, 2002), and the Pleistocene glaciation appeared to intensify the isolation between two sides of the belt where populations persisted in multiple refugia (Chen *et al*., 2008; Tian *et al*., 2009; Bai *et al*., 2010; Guo *et al*., 2014; Ye *et al*., 2017; Song *et al*., 2020). During the interglacial periods, hybrid zones were formed through range expansion as well as pollen/seed mediated gene flow. These species varied in locations and ranges of hybrid zones, possibly because the species had different degrees of gene flow and different levels of tolerance to aridity (Song *et al*., 2020).

### Three hypotheses can be ruled out as an explanation for cytonuclear discordance

Various hypotheses have been proposed to account for commonly observed cytonuclear discordance in animals and plants, including incomplete lineage sorting, positive selection, cytonuclear incompatibilities and sex‐biased dispersal (Renoult *et al*., 2009; Huang *et al*., 2014; Lee‐Yaw *et al*., 2019; Forsythe *et al*., 2020; Rose *et al*., 2021). Incomplete lineage sorting is perhaps the most commonly invoked mechanism of discordance, but it has proved difficult to distinguish from asymmetric gene flow between sexes (McKay & Zink, 2010). One crucial distinction between them is that discordance arising from asymmetric gene flow is expected to leave predictable biogeographic patterns, whereas incomplete lineage sorting cannot (Toews & Brelsford, 2012). In our case, Asian butternuts showed strong geographical patterns both in chloroplast and nuclear DNA (Figs 1, 2), implicating an insignificant role for incomplete lineage sorting. Moreover, according to theories of population genetics, a small effective population size is detrimental to the maintenance of ancestral polymorphisms. The effective population size of all the three butternut species was consistently small and had decreased rapidly since 0.5 Ma; *J. mandshurica* and *J. ailantifolia* decreased to their smallest population size (*N*_e_ ≈ 0.3 × 10^4^) after 25 ka (Fig. 3a). Therefore, we concluded that incomplete lineage sorting was insufficient to explain the cytonuclear discordance within Asian butternuts.

Lee‐Yaw *et al*. (2019) have found that positive selection on chloroplast genes may have been deeply involved in cytonuclear discordance. However, our results only provided scant evidence of positive selection on the chloroplast genome (Table 1). We identified a low level of sequence variation for our 79 protein‐code genes, and most of the fixed amino acid substitutions between the two main clades were predicted to be of little functional consequence. Only *ycf*1 may have been found subject to positive selection, as previously reported by Huang *et al*. (2014) and Lee‐Yaw *et al*. (2019). *ycf*1 has high sequence variability in seed plants (Dong *et al*., 2012) and encodes Tic214, a vital component of translocon of the inner membrane of chloroplasts (TIC) complex in Arabidopsis (Kikuchi *et al*., 2013), but little knowledge exists for other plants. At this time, positive selection seems unlikely to be a main cause for the deep divergence and steep cline of the chloroplast genome in Asian butternuts.

As regards the nuclear genome, only moderate levels of genetic differentiation were found between the two clades. Mean *F*_ST_ across the genome was 0.098 ± 0.066, similar to the value between northeast and central populations of a similarly distributed taxa, *Populus davidiana*, 0.091 (Hou *et al*., 2020). Hou *et al*. (2020) also found that very few highly differentiated regions under selection have existed between northeast and central populations. In our case, only two adjacent candidate regions harbouring two genes were identified under selection (Fig. 4). One of the two genes under selection is a *TVP38/TMEM64* family membrane protein encoding gene, which may link with the shell thickness of walnut (Bernard *et al*., 2021). The pattern of selection is consistent with the result of IBE analysis in which genetic distance is not associated with environmental distance (Fig. 5). These results converge to a limited role of selection in the formation of a narrow hybrid zone between the two clades. That only weak selection was detected for Asian butternuts may be simply due to their small effective population sizes since 0.5 Ma (Fig. 3a). All in all, the weak selection detected for Asian butternuts appears also insufficient to explain the discordance pattern.

The set of nuclear genes necessary to interact with organelles might exhibit coadaptation and the same evolution pattern with the organelle genome, whereas most of the other nuclear genes showed a different pattern, that is cytonuclear incompatibilities. Recent studies on the hybrid zone of European rabbit and killifish have found that heterogeneous introgression among loci could have been caused by cytonuclear incompatibilities at early stages of population divergence (Strand *et al*., 2012; Carneiro *et al*., 2013). In our datasets of 144 mitochondrial, 117 chloroplast and 39 dual targeted genes, Structure analyses with all the SNPs showed a similar population structure to the nuclear genome rather than the chloroplast genome (Fig. S5). Additionally, *F*_ST_ for each polymorphic site of these genes were much lower than chloroplast *F*_ST_ (Fig. 3b). As a consequence, cytonuclear incompatibility is unlikely to explain the branch‐length and geographical discordance observed in Asian butternuts. Nevertheless, owning to limited information on cytonuclear interaction genes available for nonmodel species, it is reasonable to doubt that not all interaction genes have been included in the analysis. As such, we hope our inevitably tentative analysis of cytonuclear incompatibility will provide an incentive for future explorations in nonmodel species.

### Different rates of pollen and seed gene flow most likely cause cytonuclear discordance

Given that other mechanisms of discordance have preliminarily been ruled out, sex‐biased dispersal stands out as the most probable explanation, especially for the branch‐length discordance observed for Asian butternuts. It is very easy to see that extensive pollen dispersal connects different populations well for nuclear DNA, while at the same time chloroplast DNA remains largely separated. In a similar theme, Huang *et al*. (2014) invoked chloroplast capture from an extinct poplar species to explain deep divergence in chloroplast DNA in combination with shallow nuclear divergence for balsam poplars, through elimination of incomplete lineage sorting and positive selection. Nevertheless, this hypothesis is very difficult to verify in the absence of additional evidence for signatures of ghost introgression in the nuclear genome. By contrast, Irwin (2002) put forward that deep divergence of chloroplast or mitochondrial DNA can readily emerge due to a rather small amount of dispersal limitation in continuous populations without geographic isolation. Therefore, we consider different rates of pollen and seed gene flow associated with sex‐biased dispersal as the more likely explanation of branch‐length discordance. The IBD results provided indirect evidence for differential gene flow as we found significant IBD only for nuclear genomes, but not for the chloroplast genome, which implies that the chloroplast genome was unconnected by seed gene flow. *Juglans* species are wind pollinated and pollen can travel across several kilometres, whereas fruits (walnuts) are heavy and fall in the vicinity of maternal trees, with a very small proportion successfully dispersed by scatter‐hoarding rodents (Ma *et al*., 2001, 2005). Such pronounced asymmetry in pollen vs seed dispersal is reconfirmed by our migrate analysis showing, for example, 37.91 migrants per generation from the southern to northern clade for nuclear DNA and merely 0.4 migrants per generation for chloroplast DNA.

Different rates of gene flow can also cause geographic cytonuclear discordance for Asian butternuts. In the review by Toews & Brelsford (2012), most taxa showing geographic cytonuclear discordance are groups that were isolated for long periods and experienced secondary contacts. In our case, Asian butternuts have been divided into two lineages since late Miocene and, during the period of isolation, the two lineages accumulated mutations in both their chloroplast and nuclear genomes. Upon secondary contact, the two lineages could exchange genetic material and formed a broad hybrid zones through substantial pollen gene flow, but the chloroplast genomes swapped little and formed a dividing line because of limited seed dispersal. Geographic cytonuclear discordance has also been found in *Acer mono* (Guo *et al*., 2014) and *Populus davidiana* (Song *et al*., 2020). For *Acer mono*, chloroplast DNA rather than nuclear DNA gave rise to a wider contact zone, this is likely to be as a result of extensive dispersal by winged seeds and limited pollen dispersal by insect pollination (Guo *et al*., 2014). For *Populus davidiana,* although the chloroplast DNA and nuclear DNA both formed dividing lines, the chloroplast dividing line lies further northeast than the corresponding nuclear line, which may be caused by more extensive seed dispersal (Song *et al*., 2020). For other similarly distributed taxa that have been biogeographically examined, the cytonuclear pattern remains unexplored, as most previous studies only used one set of markers, either chloroplast DNA or nuclear DNA.

Furthermore, the cytonuclear discordance in divergence and geography for *Juglans* species appeared to be intensified by invariably small historical effective population sizes, particularly since the mid‐Pleistocene (*c*. 0.5 Ma) (Fig. 3a). For example, the recent bottleneck experienced by *J. mandshurica* and *J. ailantifolia* could lead to their much lower chloroplast diversity, possibly further reinforcing the formation of the sharp dividing line of chloroplast genome. Small effective population sizes often implicate high degrees of population isolation, which in turn reinforce the difference in gene flow between pollen and seed. Irwin (2002) theoretically demonstrated that small population size and differential gene flow collaboratively contributed to cytonuclear discordance in both divergence and geography. Therefore, sexually different rates of gene flow can affect both small and large populations and can be a pervasive process underlying cytonuclear discordance for plants.

### Conclusion and significance

With the availability of full genome sequences for more systems coupled with new analysis methods, it will be interesting to see over the next few years whether reports of branch‐length and geographic cytonuclear discordance in plants grow, as they have for animals. This study offered a framework for testing sources of cytonuclear discordance and suggested that different rates of pollen and seed gene flow can be a critical force in shaping the cytonuclear discordance in plants. We advocate that future work should shift focus from documenting the prevalence of chloroplast–nuclear discordance toward testing hypotheses regarding the drivers of discordance.

## Author contributions

W‐NB and D‐YZ conceived the study. W‐NB, L‐LX and R‐MY wrote the manuscript. L‐LX, R‐MY, B‐WZ, NL, X‐RL and W‐NB performed the analyses. KL and D‐YZ contributed ideas and assisted in editing the manuscript. L‐LX and R‐MY are co‐first authors.

## Supporting information


**Fig. S1** Linkage disequilibrium (LD) decay patterns of Asian butternuts.
**Fig. S2** Schematic of demographic model analysed using fastsimcoal2 based on 23 750 SNPs of Asian butternuts.
**Fig. S3** Environmental variables used in the Gradient forest modelling.
**Fig. S4** StarBeast2 analysis of Asian butternuts based on 100 single‐copy nuclear genes.
**Fig. S5** Histograms of the Structure assignment test for 80 individuals of Asian butternuts based on the SNPs of genes interacting with chloroplast and mitochondria.
**Fig. S6** Maximum likelihood tree of 300 genes with nucleocytoplasmic interaction of Asian butternuts.
**Notes S1** Methods for extracting single‐copy nuclear genes in PhyloNet analysis.

**Notes S2** python script for conducting McDonald–Kreitman tests.
**Notes S3** Methods for environmental variables identification.
**Table S1** Details of sample locations and descriptive statistics of genome sequencing for 80 individuals of Asian butternuts.
**Table S2** Environmental variables were ordered by ranked importance.

**Table S3** Information on the 300 genes with nucleocytoplasmic interaction.
Please note: Wiley Blackwell are not responsible for the content or functionality of any Supporting Information supplied by the authors. Any queries (other than missing material) should be directed to the *New Phytologist* Central Office.

## Data Availability

Correspondence and requests for materials should be addressed to W‐NB. The data that support the findings of this study are openly available in NCBI (https://dataview.ncbi.nlm.nih.gov/object/PRJNA356989).
