# Supplementary material for: Different rates of pollen and seed gene flow cause branch‐length and geographic cytonuclear discordance within Asian butternuts
Source: New Phytol. 2021 Jul 13;232(1):388–403. doi: 10.1111/nph.17564 (PMC8519134; doi:10.1111/nph.17564)
Supplement: Supplementary file 1 — Fig. S1 Linkage disequilibrium (LD) decay patterns of Asian butternuts. Fig. S2 Schematic of demographic model analysed using fastsimcoal2 based on 23 750 SNPs of Asian butternuts. Fig. S3 Environmental variables used in the Gradient forest modelling. Fig. S4 StarBeast2 analysis of Asian butternuts based on 100 single‐copy nuclear genes. Fig. S5 Histograms of the Structure assignment test for 80 individuals of Asian butternuts based on the SNPs of genes interacting with chloroplast and mitochondria. Fig. S6 Maximum likelihood tree of 300 genes with nucleocytoplasmic interaction of Asian butternuts. Notes S1 Methods for extracting single‐copy nuclear genes in PhyloNet analysis. [file NPH-232-388-s001.pdf]

***New Phytologist* Supporting Information**

Article title: Different rates of pollen and seed gene flow cause branch-length and geographic cytonuclear discordance within Asian butternuts

Authors: Lin-Lin Xu[1†], Rui-Min Yu[1†], Xin-Rui Lin, Bo-Wen Zhang, Nan Li, Kui Lin, Da-Yong Zhang[*] and Wei-Ning Bai[*]

Article acceptance date: 13 June 2021

The following Supporting Information is available for this article:

Fig. S1 Linkage disequilibrium (LD) decay patterns of Asian butternuts (*Juglans mandshurica*, *Juglans ailantifolia* and *Juglans cathayensis*).

Fig. S2 Schematic of demographic model analyzed using *fastsimcoal2* based on 23,750 SNPs of Asian butternuts. The numbers in square brackets indicate the 95% CI of divergence time and effective population size. The grey and olive rectangles represent the most recent common ancestors of Asian butternuts and *J. mandshurica* and *J. ailantifolia*, respectively.

Fig. S3 Environmental variables used in the gradient forest modeling. *Top-ranked, uncorrelated environment variables (Pearson's $|r|$ <0 .8) and accuracy important ≥0.004.

Fig. S4 StarBEAST2 analysis of Asian butternuts based on 100 single-copy nuclear genes. Posterior probabilities are labeled on each node (red number) and the numbers in square brackets are the 95% HPD of divergence time in Ma.

Fig. S5 Histograms of the STRUCTURE assignment test for 80 individuals of Asian butternuts based on the SNPs of genes interacting with chloroplast (a), mitochondria (b) and dual (both mitochondrial and chloroplast) (c).

Fig. S6 Maximum likelihood tree of 300 genes with nucleo-cytoplasmic interaction of Asian butternuts. Bootstrap support values are labeled on each node.

Table S1 Details of sample locations and descriptive statistics of genome sequencing for 80 individuals of Asian butternuts.

Table S2 Environmental variables were ordered by ranked importance.

Table S3 Information of the 300 genes with nucleo-cytoplasmic interaction in Table S3.txt (see separate file). The first three columns are the gene ID, category and CyMIRA targeting of *Arabidopsis thaliana* and the remaining columns are the gene ID, gene length, SNP number, mean F_{ST}, maximum F_{ST}, minimum F_{ST} of *J. mandshurica*.

Notes 1 Methods for extracting single copy nuclear genes in PhyloNet analysis.

Notes 2 Python script for conducting McDonald-Kreitman tests (see separate file).

Note 3 Methods for environmental variables identification.

Fig. S1 Linkage disequilibrium (LD) decay patterns of Asian butternuts (*J. mandshurica, J. ailantifolia* and *J. cathayensis*).

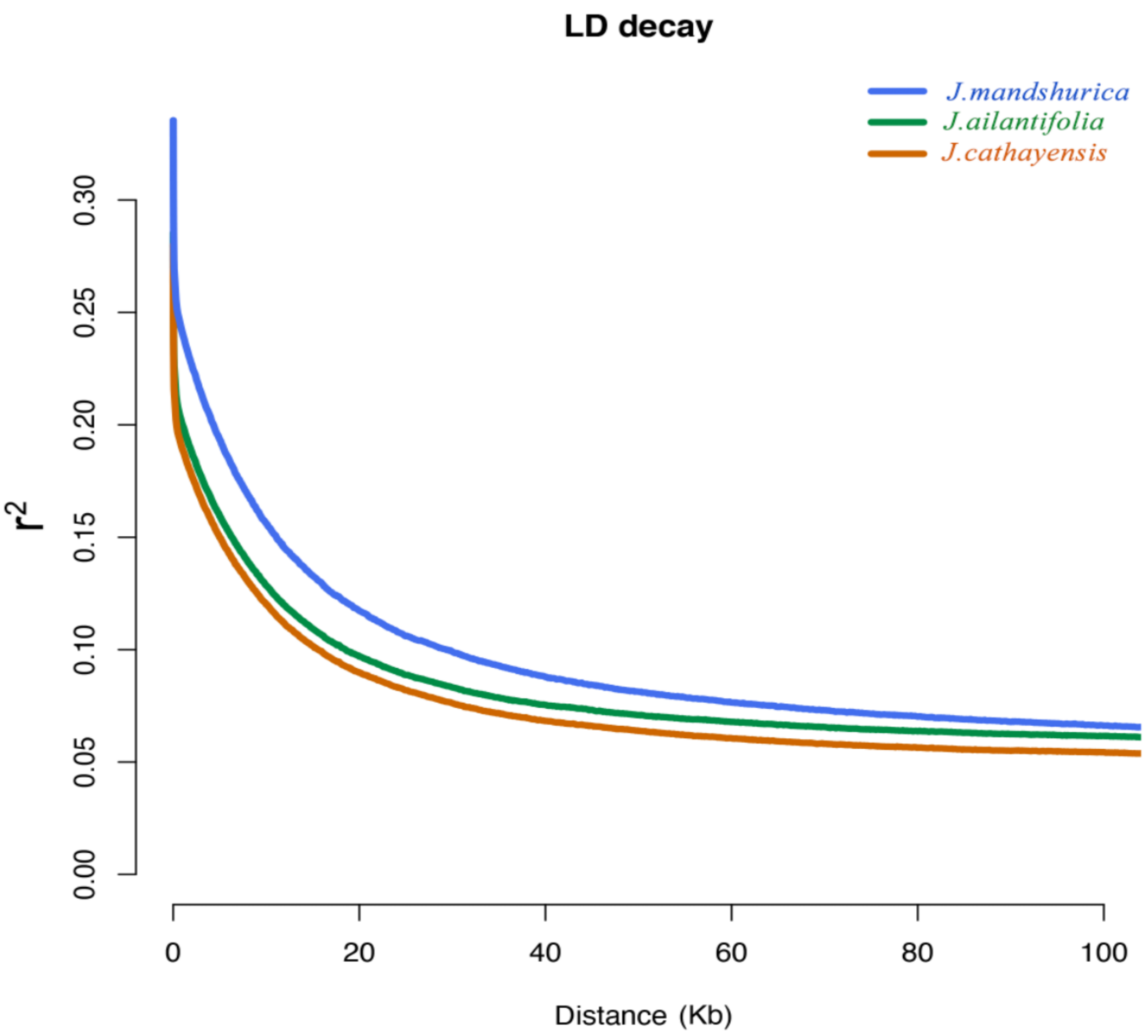

Fig. S2 Schematic of demographic model analyzed using fastsimcoal2 based on 23,750 SNPs of Asian butternuts. The numbers in square brackets indicate the 95% CI of divergence time and effective population size. The grey and olive rectangles represent the most recent common ancestors of Asian butternuts and *J. mandshurica* and *J. ailantifolia*, respectively.

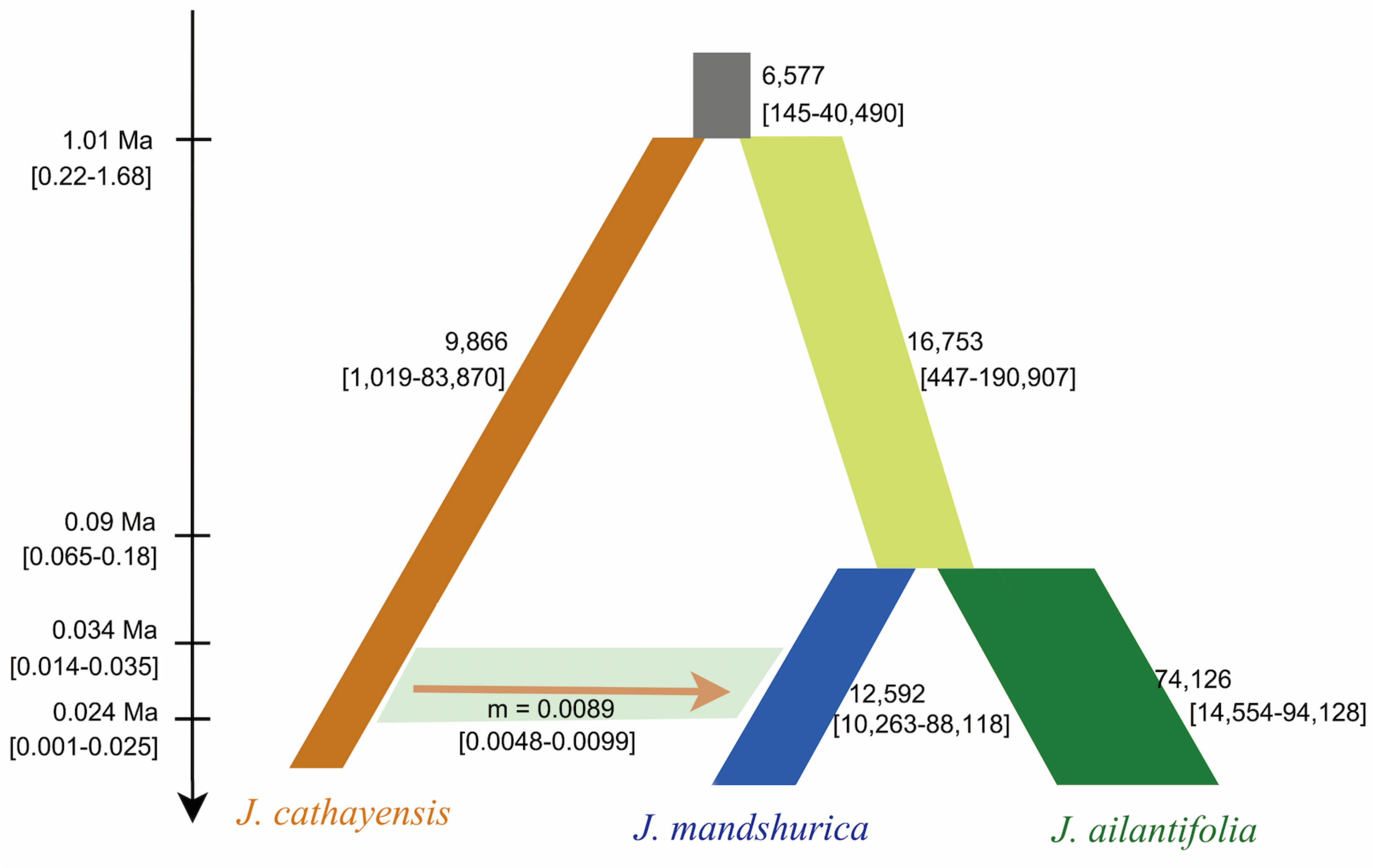

Fig. S3 Environmental variables used in the gradient forest modeling. *Top-ranked, uncorrelated environment variables (Pearson's |*r*| <0 .8) and accuracy important ≥0.004.

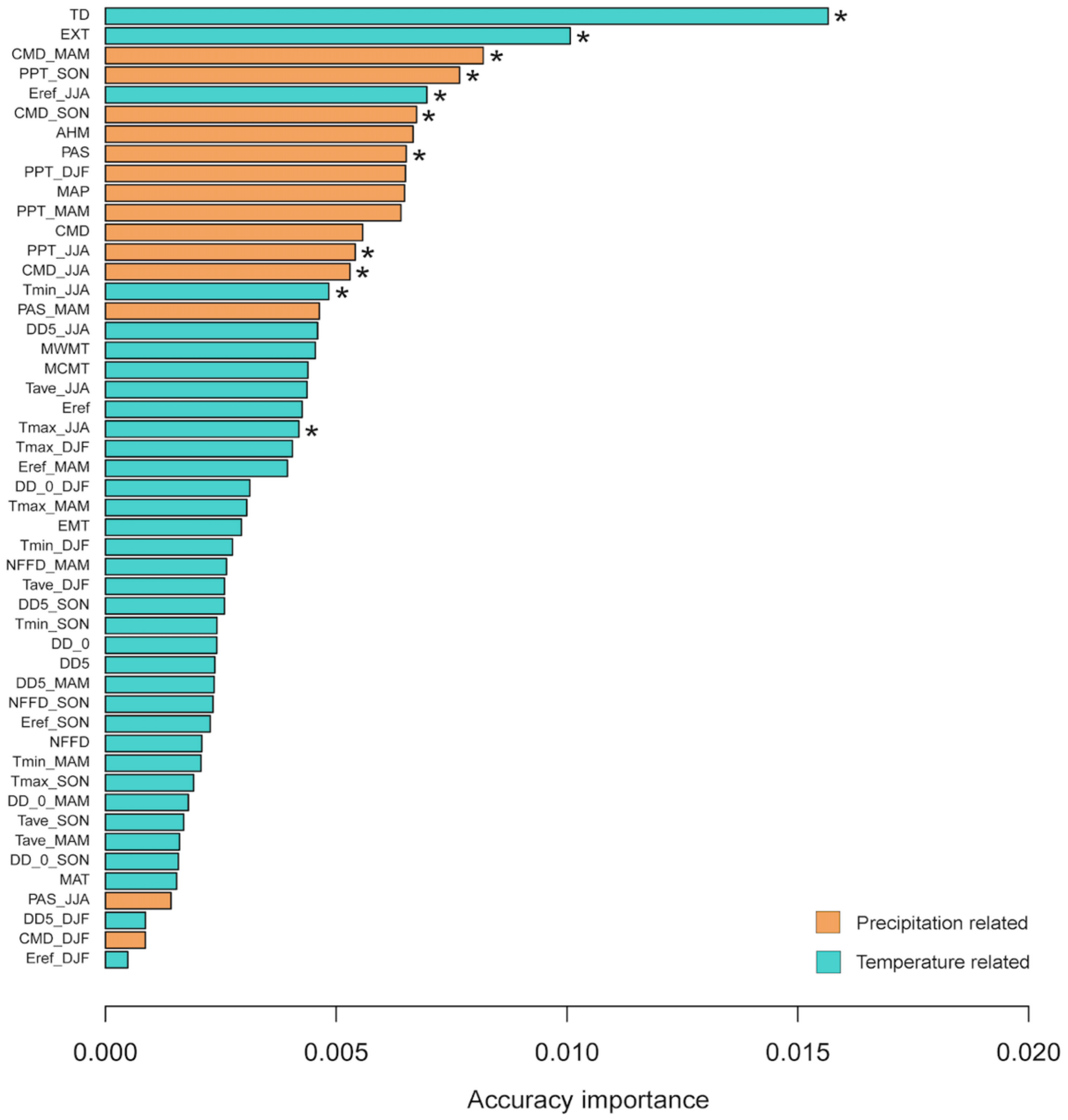

Fig. S4 StarBEAST2 analysis of Asian butternuts based on 100 single-copy nuclear genes. Posterior probabilities are labeled on each node (red number) and the numbers in square brackets are the 95% HPD of divergence time in Ma.

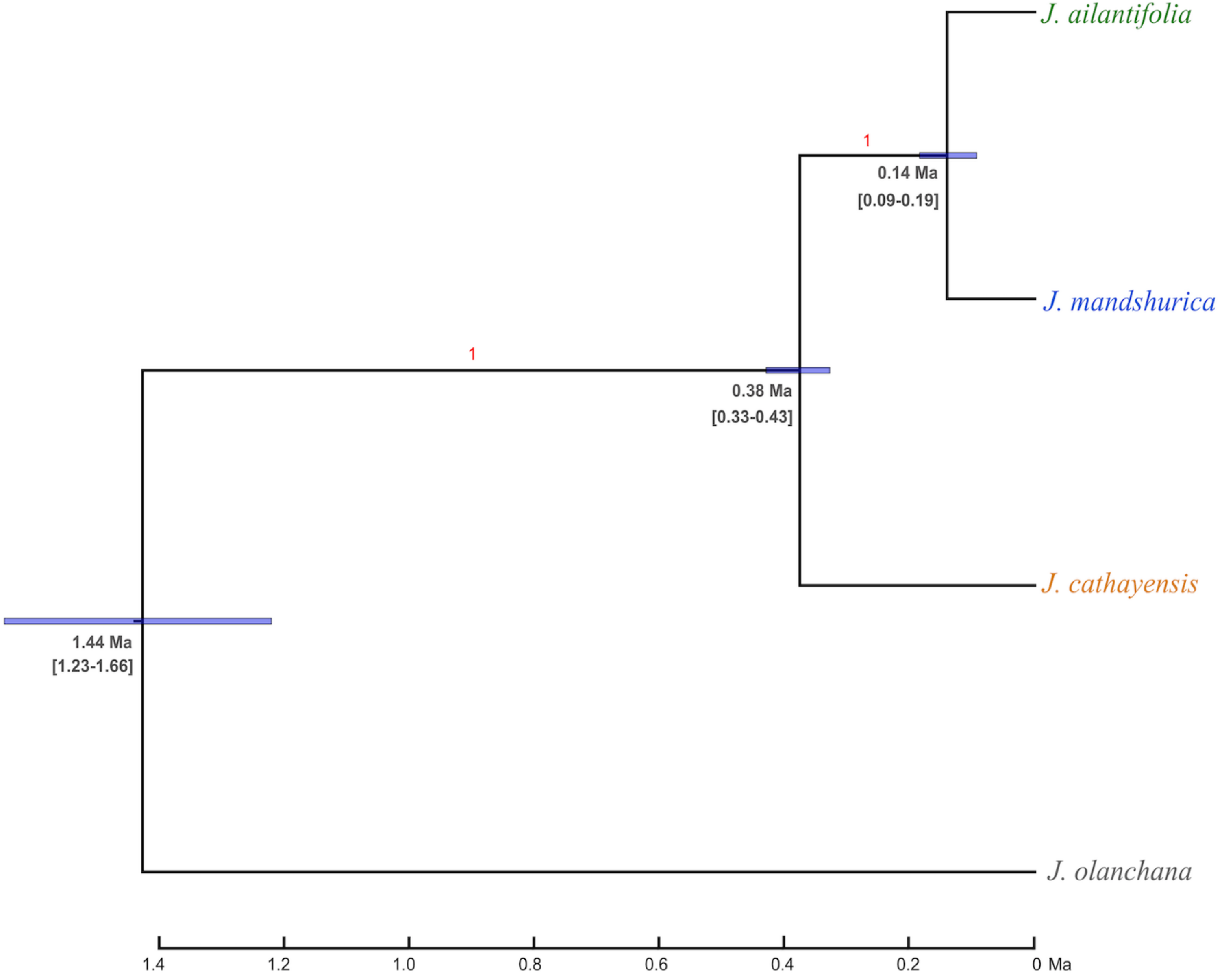

Fig. S5 Histograms of the STRUCTURE assignment test for 80 individuals of Asian butternuts based on the SNPs of genes interacting with chloroplast (a), mitochondria (b) and dual (both mitochondrial and chloroplast) (c).

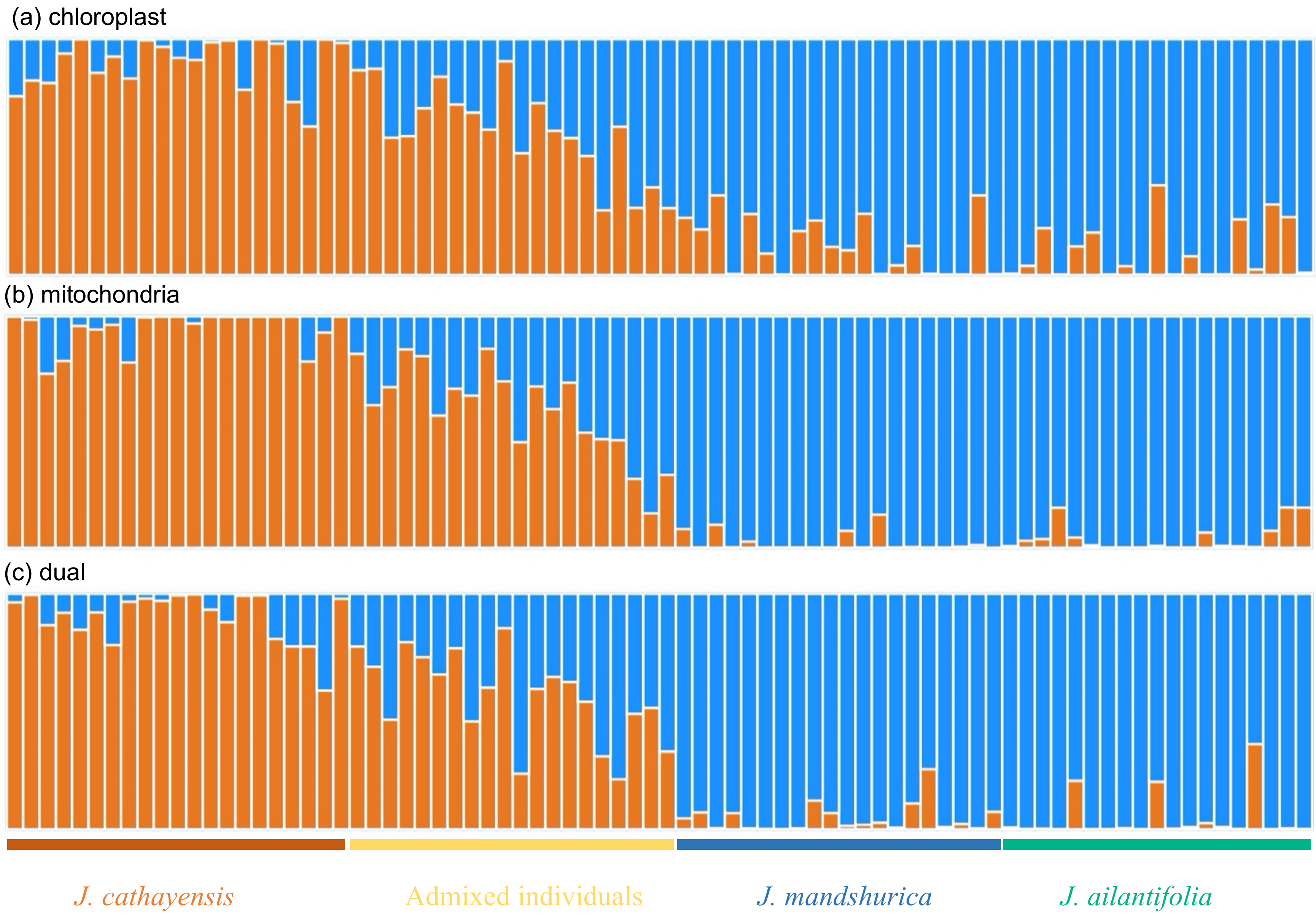

Fig. S6 Maximum likelihood tree of 300 genes with nucleo-cytoplasmic interaction of Asian butternuts. Bootstrap support values are labeled on each node.

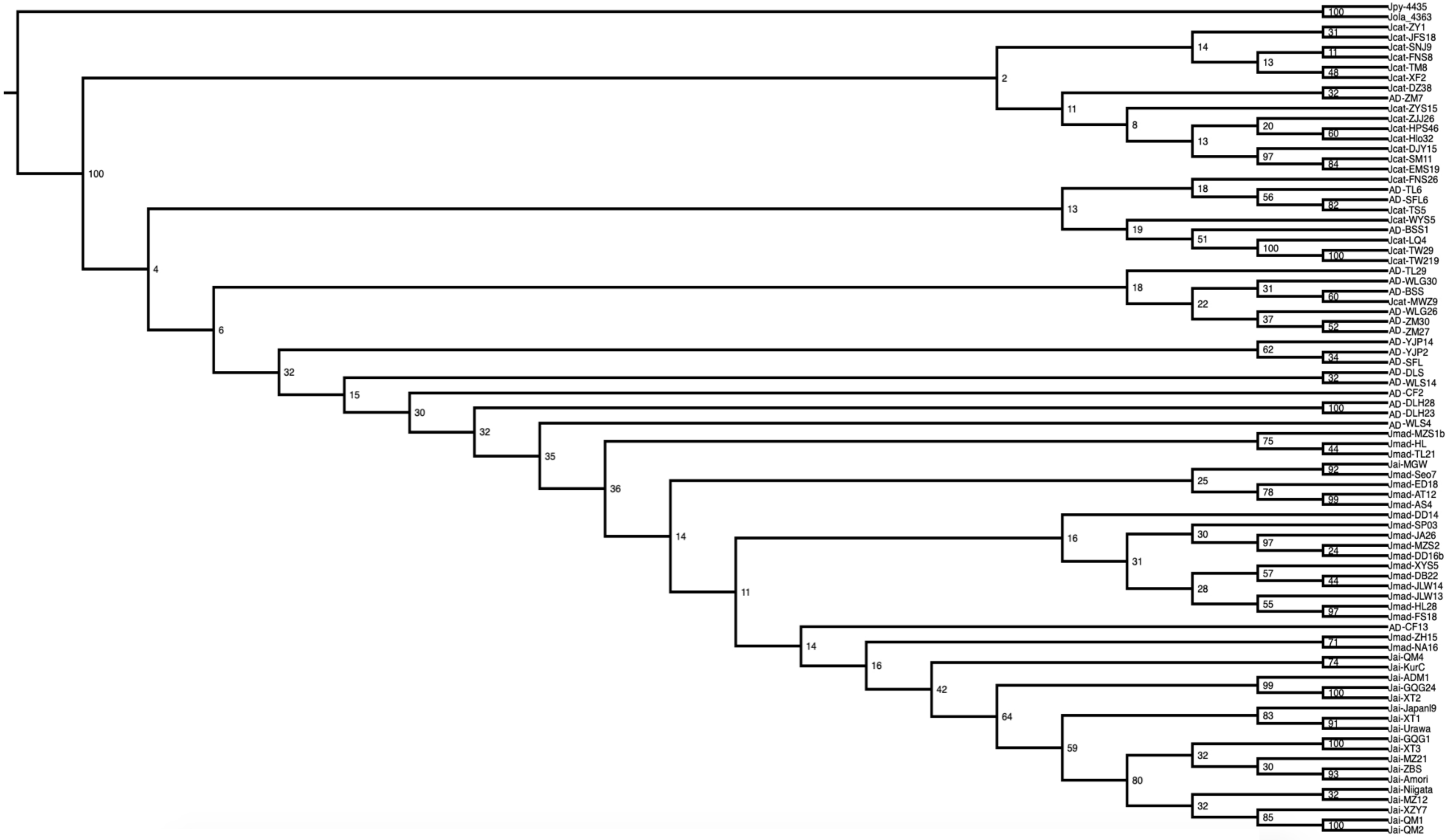

Table S1. Details of sample locations and descriptive statistics of genome sequencing for 80 individuals of Asian butternuts.

Population	Sample	Location	Latitude	Longitude	Depth	Coverage	CpDNA haplotype
J. ailantifolia	JA-Amori	Japan	40.82	140.82	37.80	94.85%	JA_1
	JA-GQG24	Japan	36.26	139.01	31.54	94.76%	JA_2
	JA-Japanl9	Japan	36.31	138.98	35.44	94.83%	JA_2
	JA-MGW	Japan	35.10	136.55	35.54	94.23%	JA_2
	JA-Urawa	Japan	35.51	139.39	49.30	96.17%	JA_2
	JA-Niigata	Japan	37.90	139.02	33.88	95.15%	JA_2
	JA-QM1	Japan	35.79	139.24	28.63	94.64%	JA_2
	JA-QM2	Japan	35.79	139.24	33.24	94.73%	JA_2
	JA-QM4	Japan	35.79	139.24	31.88	94.91%	JA_2
	JA-MZ11	Japan	42.58	141.33	13.22	94.16%	JA_2
	JA-MZ12	Japan	42.58	141.33	31.59	95.06%	JA_2
	JA-MZ21	Japan	42.58	141.33	29.37	94.76%	JA_2
	JA-XT1	Japan	38.27	141.03	36.55	95.07%	JA_2
	JA-XT2	Japan	38.27	141.03	30.97	94.71%	JA_2
	JA-XT3	Japan	38.27	141.03	32.27	94.97%	JA_2
	JA-GQG1	Japan	36.26	139.07	34.31	94.84%	JA_1
	JA-XZY7	Japan	42.86	141.32	38.76	95.12%	JA_2

	JA-ZBS	Japan	36.22	140.10	37.97	95.15%	JA_2
	JA-ADM1	Japan	35.67	139.01	33.61	94.98%	JA_2
	Jai-KurC	Japan	37.90	139.07	41.69	94.77%	JA_2
J. mandshurica	JM-DB22	Daban Liaoning	41.89	121.77	39.71	95.08%	JM_1
	JM-MZS1b	Maoershan Heilongjiang	45.4	127.65	26.62	94.89%	JM_1
	JM-MZS2	Maoershan Heilongjiang	45.4	127.65	43.70	95.24%	JM_1
	JM-NA16	Ningan Jilin	44.35	129.53	32.66	95.02%	JM_2
	JM-JA26	Jian Jilin	41.18	126.23	35.58	95.14%	JM_1
	JM-SP03	Siping Jilin	43.17	124.35	31.59	95.31%	JM_1
	JM-HL	Hulin Heilongjiang	45.86	133.00	43.89	95.40%	JM_1
	JM-HL28	Hulin Heilongjiang	45.86	133.00	35.80	95.24%	JM_1
	JM-ZH15	Zhuanghe Liaoning	39.70	122.97	43.45	94.88%	JM_1
	JM-AT12	Antu Jilin	43.11	128.90	22.70	93.58%	JM_1
	JM-ED18	Erdao Jilin	42.49	128.24	26.62	94.94%	JM_1
	JM-DD14	Dandong Liaoning	40.30	124.16	47.36	95.21%	JM_1
	JM-DD16b	Dandong Liaoning	40.30	124.16	31.52	94.80%	JM_1
	JM-FS18	Fushun Liaoning	41.96	124.25	27.33	95.03%	JM_1
	JM-TL21	Tieli Heilongjiang	47.98	128.08	20.70	94.59%	JM_1
	JM-AS4	Anshan Liaoning	41.02	123.2	30.81	94.89%	JM_1
	JM-Seo7	Korea	37.45	127.08	37.45	95.04%	JM_3

	JM-JLW13	Korea	37.43	128.56	24.15	94.93%	JM_2
	JM-JLW14	Korea	37.43	128.56	33.42	95.25%	JM_2
	JM-XYS5	Korea	38.17	128.49	42.44	95.43%	JM_2
J. cathayensis	JC-DZ38	Dongzhai Henan	30.9	114.27	29.19	94.75%	JC_6
	JC-ZYS15	Ziyunshan Hunan	26.69	111.1	26.69	94.55%	JC_2
	JC-FNS26	Funiushan Henan	33.32	111.50	31.62	94.84%	JC_11
	JC-FNS8	Funiushan Henan	33.32	111.50	32.63	94.65%	JC_11
	JC-HPS46	Haopingsi Shaanxi	34.07	107.70	27.36	94.60%	JC_10
	JC-WYS5	Wuyishan Jiangxi	27.72	117.71	35.88	94.62%	JC_4
	JC-JFS18	Jiefushan Hunan	27.25	111.72	27.15	94.38%	JC_1
	JC-LQ4	Longquan Zhejiang	27.87	119.15	27.50	93.90%	JC_5
	JC-TM8	Tianmushan Zhejiang	30.36	119.45	29.04	94.55%	JC_7
	JC-SNJ9	Shennongjia Hubei	31.40	109.93	24.99	94.53%	JC_9
	JC-TS5	Tianshui Gansu	34.31	106.12	30.89	94.76%	JC_10
	JC-ZY1	Zunyi Guizhou	28.22	107.16	36.99	94.65%	JC_13
	JC-TW219	Taiwan	24.39	121.35	34.59	94.48%	JC_3
	JC-TW29	Taiwan	24.39	121.35	34.55	94.23%	JC_3
	JC-ZJJ26	Zhangjiajie Hunan	29.08	110.29	42.78	94.35%	JC_8
	JC-DJY15	Dujiangyan Sichuan	30.96	103.38	38.86	94.57%	JC_15
	JC-EMS19	Ermeishan Sichuan	29.58	103.36	28.17	94.73%	JC_16

	JC-Hlo32	Huanglong Sichuan	32.69	104.06	39.39	94.73%	JC_10
	JC-SM11	Shimian Sichuan	29.21	102.38	37.16	94.86%	JC_14
	JC-MWZ9	Mawuzhai Shanxi	35.78	113.27	34.35	94.79%	JC_11
	JC-XF2	Xianfeng Hubei	29.40	109.11	43.17	94.67%	JC_12
**Admixed individuals**	AD-CF2	Chifeng Neimenggu	43.28	117.42	28.51	94.93%	JM_1
	AD-CF13	Chifeng Neimenggu	43.28	117.42	39.55	94.92%	JM_1
	AD-DLS	Donglingshan Beijing	39.97	115.42	29.55	93.03%	JM_1
	AD-DLH23	Daluhua Liaoning	41.51	121.62	42.32	95.21%	JM_1
	AD-DLH28	Daluhua Liaoning	41.51	121.62	29.22	95.10%	JM_1
	AD-SFL6	Shifoling Hebei	39.97	115.35	28.39	95.02%	JM_1
	AD-SFL	Shifoling Hebei	39.97	115.35	32.13	94.78%	JM_1
	AD-TL6	Tuoliang Shanxi	38.73	113.80	32.59	92.32%	JC_11
	AD-TL29	Tuoliang Shanxi	38.73	113.80	35.48	94.70%	JC_11
	AD-YJP14	Yangjiaping Hebei	39.97	115.39	34.38	94.83%	JM_1
	AD-YJP2	Yangjiaping Hebei	39.97	115.39	35.61	94.87%	JM_1
	AD-ZM27	Zhangmo Hebei	37.35	114.17	39.10	94.75%	JC_11
	AD-ZM30	Zhangmo Hebei	37.35	114.17	33.27	94.82%	JC_11
	AD-ZM7	Zhangmo Hebei	37.36	114.22	27.54	94.85%	JC_11
	AD-BSS	Baishishan Hebei	39.37	114.69	28.97	91.99%	JC_11
	AD-BSS1	Baishishan Hebei	39.37	114.69	27.51	94.91%	JC_11

AD- WLG30	Wulonggou Hebei	39.49	114.93	32.69	95.02%	JC_11
AD-WLG26	Wulonggou Hebei	39.49	114.93	21.59	95.03%	JM_1
AD-WLS4	Wulingshan Hebei	40.96	117.93	28.00	95.12%	JM_1
AD-WLS14	Wulingshan Hebei	40.96	117.93	34.18	95.13%	JM_1

Table S2. Environmental variables used in our study.

Category	Code	Description
Annual	TD	Temperature difference between MWMT and MCMT, or continentality (°C)
Annual	EXT	Extreme maximum temperature over 30 years
Seasonal	CMD_MAM	Hargreaves climatic moisture deficit in March, April and May
Seasonal	PPT_SON	Precipitation in September, October and November
Seasonal	Eref_JJA	Hargreaves reference evaporation in June, July and August
Seasonal	CMD_SON	Hargreaves climatic moisture deficit in September, October and November
Annual	AHM	Annual heat: moisture index ((MAT+10)/(MAP/1000))
Annual	PAS	Precipitation as snow (mm) between August in previous year and July in current year
Seasonal	PPT_DJF	Precipitation in December, January and February
Annual	MAP	Mean annual precipitation (mm)
Seasonal	PPT_MAM	Precipitation in March, April and May
Annual	CMD	Hargreaves climatic moisture deficit
Seasonal	PPT_JJA	Precipitation in June, July and August
Seasonal	CMD_JJA	Hargreaves climatic moisture deficit in June, July and August
Seasonal	Tmin_JJA	Minimum temperature in June, July and August
Seasonal	PAS_MAM	Precipitation as snow (mm) between August in previous year and July in current year
Seasonal	DD5_JJA	Degree-days above 5°C in June, July and August

Annual	MWMT	Mean warmest month temperature (°C)
Annual	MCMT	Mean coldest month temperature (°C)
Seasonal	Tave_JJA	Mean temperature in June, July and August
Annual	Eref	Hargreaves reference evaporation
Seasonal	Tmax_JJA	Maximum temperature in June, July and August
Seasonal	Tmax_DJF	Maximum temperature in December, January and February
Seasonal	Eref_MAM	Hargreaves reference evaporation in March, April and May
Seasonal	DD_0_DJF	Degree-days below 0°C in December, January and February
Seasonal	Tmax_MAM	Maximum temperature in March, April and May
Annual	EMT	Extreme minimum temperature over 30 years
Seasonal	Tmin_DJF	Minimum temperature in December, January and February
Seasonal	NFFD_MAM	The number of frost-free days in March, April and May
Seasonal	Tave_DJF	Mean temperature in December, January and February
Seasonal	DD5_SON	Degree-days above 5°C in September, October and November
Seasonal	Tmin_SON	Minimum temperature in September, October and November
Annual	DD_0	Degree-days below 0°C
Annual	DD5	Degree-days above 5°C
Seasonal	DD5_MAM	Degree-days above 5°C in March, April and May
Seasonal	NFFD_SON	The number of frost-free days in September, October and November
Seasonal	Eref_SON	Hargreaves reference evaporation in September, October and November
Annual	NFFD	The number of frost-free days
Seasonal	Tmin_MAM	Minimum temperature in March, April and May
Seasonal	Tmax_SON	Maximum temperature in September, October and November
Seasonal	DD_0_MAM	Degree-days below 0°C in March, April and May
Seasonal	Tave_SON	Mean temperature in September, October and November

Seasonal	Tave_MAM	Mean temperature in March, April and May
Seasonal	DD_0_SON	Degree-days below 0°C in September, October and November
Annual	MAT	Mean annual temperature (°C)
Seasonal	PAS_JJA	Precipitation as snow (mm) between August in previous year and July in current year
Seasonal	DD5_DJF	Degree-days above 5°C in December, January and February
Seasonal	CMD_DJF	Hargreaves climatic moisture deficit in December, January and February
Seasonal	Eref_DJF	Hargreaves reference evaporation in December, January and February

Notes 1. Orthologous single copy nuclear genes extraction.

First, we mapped short reads of 30 individuals of Asian butternuts and an outgroup (*J. olanchana*) to *J. mandshurica* reference genome using the BWA-MEM algorithm in BWA v. 0.7.12. The consensus sequence was called using the SAMTOOLS v.1.3 suite utilizing the samtools mpileup, bcftools and vcfutils.pl (varFilter) pipeline. To ensure a consensus sequence of high confidence, we set the software parameters -C to 50, -q to 20, -Q to 20, and removed the insertion and deletion as well as variation within 3bp around them. Minimum read depth (-d) and maximum read depth (-D) were set to 10 and 60 and marked the sites lower than 10× and higher than 60× as missing data "N". Consensus genomes were built based on the above SNPs for each individual (Nielsen *et al.*, 2011). CDS were extracted from the consensus genome, based on the annotation and orthologous analysis by Zhang *et al.* (2019).

Next, we used OrthoFinder v0.4.0 (Emms & Kelly, 2015) and ProteinOrtho v6.0.23 (Lechner *et al.*, 2014) to distinguish orthologous single-copy genes of seven reference genome, and obtained 4,006 and 3,312 genes, respectively. A total of 1,645 genes were remained in the two datasets. The genes whose length are between 300 bp to 1,000 bp and more than 50 kb far apart and less than 50% of missing match were remained. Finally, we obtained 1,622 genes for butternuts. Protein sequences of each gene were aligned by MAFFT v. 7.273 (Katoh *et al.*, 2002) , and corresponding codons were assigned into codon alignments by PAL2NAL v. 14 (Suyama *et al.*, 2006).

Notes 2. Python script (Caculate_mktest.py) for conducting McDonald-Kreitman tests (see separate file).

Notes 3. Environmental variables identification for Gradient forest (GF) analyses.
For each sampling location, we used a high-resolution climate database, climateAP (Wang *et al.*, 2017) to choose 49 variables with known impacts on plant survival and development, including 14 annual and 35 seasonal environmental variables (Table S2) (Jia *et al.*, 2020). We performed GF analyses to identify the environmental variables that best explained the distribution of genetic diversity using the R package Gradient forest (Ellis *et al.*, 2012). To be sure the SNPs are independent, we kept only one SNP per 20 kb for GF analyses. We used 1,000 regression trees per SNP to fit GF model while keeping all the parameters set at default values. After evaluating the accuracy importance and Pearson pairwise correlations among these variables, 11 variables with absolute value of Pearson correlation coefficient (r) $\leq$0.8 and accuracy important $\geq$0.004 were retained for the following analysis (Fig. S3). The 11 selected climate variables were used to calculate environmental distances by first scaling and centering the variables to account for differences in magnitude, then calculating pairwise Euclidean differences between sites.

Ellis N, Smith SJ, Pitcher CR. 2012. Gradient forests: calculating importance gradients on physical predictors. *Ecology* **93**: 156-168.

Emms DM, Kelly S. 2015. OrthoFinder: solving fundamental biases in whole genome comparisons dramatically improves orthogroup inference accuracy. *Genome Biology* **16:** doi:10.1186/s13059-015-0721-2.

Jia KH, Zhao W, Maier PA, Hu XG, Jin YQ, Zhou SS, Jiao SQ, El-Kassaby YA, Wang TL, Wang XR, et al. 2020. Landscape genomics predicts climate change-related genetic offset for the widespread *Platycladus orientalis* (Cupressaceae). *Evolutionary Applications* **13**: 665-676.

Katoh K, Misawa K, Kuma K, Miyata T. 2002. MAFFT: a novel method for rapid multiple sequence alignment based on fast Fourier transform. *Nucleic Acids Research* **30**: 3059-3066.

Lechner M, Hernandez-Rosales M, Doerr D, Wieseke N, Thevenin A, Stoye J, Hartmann RK, Prohaska SJ, Stadler PF. 2014. Orthology Detection Combining Clustering and Synteny for Very Large Datasets. *PLos One* **9**: e105015.

Nielsen R, Paul JS, Albrechtsen A, Song YS. 2011. Genotype and SNP calling from next-generation sequencing data. *Nature Reviews Genetics* **12**: 443-451.

Suyama M, Torrents D, Bork P. 2006. PAL2NAL: robust conversion of protein sequence alignments into the corresponding codon alignments. *Nucleic Acids Research* **34**: W609-W612.

Wang T, Wang G, Innes JL, Seely B, Chen B. 2017. ClimateAP: an application for dynamic local downscaling of historical and future climate data in Asia Pacific. *Frontiers of Agricultural Science and Engineering* **4**: 448-458.

Zhang BW, Xu LL, Li N, Yan PC, Jiang XH, Woeste KE, Lin K, Renner SS, Zhang DY, Bai WN. 2019. Phylogenomics Reveals an Ancient Hybrid Origin of the Persian Walnut. *Molecular Biology and Evolution* **36**: 2451-2461.
